# Lack of evidence supporting a role for DPP6 sequence variants in Alzheimer’s disease in the European American population

**DOI:** 10.1007/s00401-021-02271-w

**Published:** 2021-02-16

**Authors:** Laxmi Kirola, John P. Budde, Fengxian Wang, Joanne Norton, John C. Morris, NIA-LOAD family study group, NCRAD, the ADSP project, Carlos Cruchaga, Maria Victoria Fernández

**Affiliations:** 1 Department Psychiatry, Washington University School of Medicine (WUSM), 660 S. Euclid Ave. B8134, St. Louis, MO 63110 USA; 2 Hope Center for Neurological Disorders, WUSM, 660 S. Euclid Ave. B8111, St. Louis, MO 63110 USA; 3 NeuroGenomics and Informatics Center, Washington University School of Medicine, 660 S. Euclid Ave. B8134, St. Louis, MO USA; 4 Knight Alzheimer Disease Research Center, WUSM, 4488 Forest Park Ave, St. Louis, MO 63108 USA

Using linkage analysis in a large Dutch early onset AD (EOAD) family, Rademakers et al. identified a candidate region chromosome 7q36 [7]. Follow-up studies of this region revealed a chromosomal inversion disrupting the coding sequence of *DPP6* in the Dutch family, as well as several rare non-synonymous variants in a large EOAD Belgian cohort [2, 7]. *DPP6* encodes a transmembrane protein, predominantly expressed in the brain, which binds to potassium channel Kv4.2 and regulates its gate activity, dendritic excitability and plasticity of hippocampal pyramidal neurons [6]. In vitro modeling showed reduced *DPP6* expression in brain tissue of missense variant carriers and loss of protein which causes hyperexcitability and behavioral alterations in Dpp6-KO mice.

Here, we investigate whole exome sequence data (WES) for the potential association of coding variants present in *DPP6* with AD, in three European American cohorts: the Familial Alzheimer Sequencing (FASe) project [5], an unrelated EOAD, and the unrelated Alzheimer Disease Sequencing Project (ADSP—pht003392.v7.p4) [1]. Cryptic relatedness and population admixture were performed and only non-Hispanic whites (according to the first two genetic principal components (PC) using Hapmap as reference panel) were kept for further analyses (Table 1).

**Table 1:** Demographic characteristics of each of the cohorts employed in this study

Cohort	Status	*N*	%Fe	%APOE ε4	Age (*Χ* ± SD)
FASe	CA	1,212	63.61	69.66	72.71 ± 9.51
	CO	341	56.89	51.24	80.52 ± 9.70
EOAD	CA	1,385	51.91	67.87	60.39 ± 2.91
	CO	3,864	61.05	61.05	91.27 ± 8.01
ADSP	CA	5,656	57.00	42.35	75.50 ± 9.30
	CO	4,601	59.00	14.00	87.20 ± 8.20

*CA* cases, *CO* controls, *%Fe* percentage of female, *%APOE4* percentage of APOE ε4

We examined five isoforms (ENST00000377770, ENST00000406326, ENST00000406326, ENST00000332007, ENST00000427557) of *DPP6* for annotation purposes. We performed single variant logistic regression analysis using PLINK 1.9 [3], and two burden tests: (i) non-synonymous rare variants with MAF ≤ 1%; (ii) non-synonymous variants with a CADD ≥ 20 using SKAT-O [8]. We used sex and the first three genetic PCs (PC1, PC2 and PC3) as covariates in all analyses.

We identified 15 *DPP6* variants in FASe, 32 in EOAD and 143 in ADSP (Supplementary Table 1). No single variant was significant in any of the cohorts examined. We identified 42 and 3 nonsynonymous variants with a MAF ≤ 1% (Supplementary Table 2), and 39 and 2 variants with a CADD ≥ 20 in the ADSP and EOAD cohort (Supplementary Table 3), respectively, SKAT-O tests were non-significant (Table 2). For the FASe cohort, we only detected one rare nonsynonymous variant with a CADD ≥ 20 so this cohort was non-informative for gene-burden purposes.

**Table 2:** Gene burden analysis on *DPP6* variants in EOAD and ADSP datasets

Cohorts	Gene set	*N*	cummOR	SKAT-O
EOAD	MAF ≤ 1%	3	5.32	0.47
	CADD ≥ 20	2	NA	0.26
ADSP	MAF ≤ 1%	37	0.94	0.72
	CADD ≥ 20	38	0.98	0.87

*MAF* minor allele frequency, *CADD* combined annotation-dependent depletion, *N* number of variants included in the burden analysis, *cummOR* cummulative Odds Ratio

Cacace et al. reported 7 pathogenic variants within Exon1 of *DPP6*, and 13 variants in the extracellular domain. We found 8 of the 25 variants reported [2] in the ADSP cohort (p.Pro229Thr, p.Arg274His, p.Arg322His, p.His357Arg, p.Lys570Asn, p.Lys571Gln, p.Ala655Thr, p.Ala778Thr) and one of those (p.Ala655Thr) in the EOAD cohort (Supplementary Table 1). We did not detect any of the variants reported by Cacace et al. on Exon1, regardless of the isoform examined.

In vitro modeling for variants p.Glu208Gln (found in a Frontotemporal Dementia patient), p.Arg274His, p.Arg322His, p.His357Arg (identified in AD patients) and p.Pro509Arg (found in a primary progressive aphasia patient) found that these variants destabilize the protein leading to a reduced level on the plasma membrane [2]. Only the p.His357Arg was observed with the same direction of effect (present only in cases) in both [2] and the ADSP (Supplementary Table 1). We found p.Arg274Hist in one CO and p.Arg322Hist in one CA and one CO of the ADSP, but we did not identify carriers for either p.E208Q or p.P509Q.

To summarize, we performed single variant and burden analyses for *DPP6* in three cohorts of non-Hispanic white individuals: FASe, EOAD and ADSP. Neither the recently reported *DPP6* variants [2] nor any other rare variants found in our study would confer risk to AD in European Americans, despite our cohorts (FASe, EOAD, and ADSP) were larger than that of [2] (CA = 558 and CO = 775), and we had enough statistical power (96.4%, α = 0.05, MAF = 0.01, OR = 2.00) to replicate their findings. Cacace et al. [2] reported a high burden of rare variants in *DPP6* which could be better explained with a possible population isolation effect of *DPP6* variants in Dutch population [7]. This correlation between rarity of a gene with population specificity has been previously reported for other AD risk loci [4]. Nonetheless, further studies should be conducted to clarify the real implication of this gene in AD in general, but also towards other neurodegenerative diseases, given that (i) Cacace et al. identified carriers of missense variants in FTD and PSP patients (ii) the functional studies from Cacace et al. that indicated that the missense mutations did alter the protein structure; and (iii) we only examined the exonic regions and some of the reported variants by Cacace et al. correspond to intronic structural variants.

## Supplementary Information

Below is the link to the electronic supplementary material.

Supplementary file1 (XLSX 92 KB)
